# Epilepsy EEG Signal Classification Algorithm Based on Improved RBF

**DOI:** 10.3389/fnins.2020.00606

**Published:** 2020-06-23

**Authors:** Dongmei Zhou, Xuemei Li

**Affiliations:** ^1^College of Information Science and Technology, Chengdu University of Technology, Chengdu, China; ^2^Xijing Hospital, Air Force Medical University, Xi'an, China

**Keywords:** epilepsia, EEG signal, convolution neural network, RBF, one-against-one

## Abstract

Epilepsy is a chronic recurrent transient brain dysfunction syndrome. It is characterized by recurrent epilepsy caused by abnormal discharge of brain neurons. Epilepsy is one of the common diseases in nervous system. The analysis of EEG signals is a hot topic in current research. In order to solve the problem of epileptic EEG signals classification accurately, we carry out in-depth research on epileptic EEG signals, analyze features from linear and non-linear perspectives, input them into the improved RBF model to dynamically extract effective features, and introduce one against one strategy classifier to reduce the probability of error classification. Experiments show that the proposed algorithm has strong robustness and high epileptic signal recognition rate.

## Introduction

Epilepsy is a transient brain dysfunction caused by sudden abnormal over discharge of brain neurons, which has a high incidence rate (Jiang et al., 2020). The detection and recognition of EEG signal are the most important means to diagnose epilepsy. The method of multi-feature extraction and intelligent recognition has been applied to the recognition of epileptic EEG signals (Ojha et al., 2020). Guo et al. (2010) realizes EEG signal classification based on intelligent network. Faust et al. (2010) analyzes EEG information of epilepsy in frequency domain. Wang et al. (2011) establishes wavelet model to classify signals. Hubsch et al. (2011) establishes a model for EEG analysis from the perspective of video. Chua et al. (2011) uses high-order features to realize epilepsy signal recognition. Kumar et al. (2012) proposes the classification of epileptic signals by relative wave energy and wave entry. Tzallas et al. (2012) reviews the history of epileptic brain signal recognition. Khan et al. (2012) uses multi-dimensional wavelet transform to detect epileptic signals. Murugavel et al. (2013) establishes SVM classifier to realize EEG classification. Zhu et al. (2013) analyzes the distribution of EEG signals from the perspective of energy. Wang et al. (2013) extracts fractal features for EEG analysis. Kumar et al. (2014) analyzes EEG based on fuzzy set. Yuan et al. (2014) uses different kernel functions to classify epileptic signals. Xie and Krishnan (2014) introduces sliding window to block EEG analysis. Kaya (2015) analyzes EEG signals based on local binary patterns. Faust et al. (2015) uses computer-aided means to identify EEG signals. Martis et al. (2015) uses multiple frequency bands to analyze EEG signals of epilepsy. Djemili et al. (2016) introduces artificial mode to distinguish epileptic signals from ordinary signals. Al Ghayab et al. (2016) extracts features from EEG signals by random sampling. Murugavel and Ramakrishnan (2016) establishes SVM classifier to classify EEG signals. Li et al. (2017) extracts the non-linear structure of EEG to realize the automatic identification of EEG signals. Tibdewal et al. (2017) carries out research on the basis of multichannel epileptic EEG signals. Sharma and Pachori (2017) establishes a model from the time and space dimensions for analysis. Sharma et al. (2018) uses iterative filtering to recognize EEG signals. Prabhakar and Rajaguru (2018) establishes AdaBoost classifier to realize multi-dimensional EEG analysis. Zhou et al. (2018) introduces CNN to analyze EEG signals. Buettner et al. (2019) extracts higher-order features for EEG analysis. Raghu et al. (2019) realizes the signal recognition of epileptic seizure based on matrix terminator. Hossain et al. (2019) establishes a deep learning network to visualize brain imaging. Parija et al. (2020) establishes a model from the perspective of multi-core to analyze EEG. Li et al. (2020) analyzes the instantaneous signal strength. Seo et al. (2020) establishes a dynamic model to recognize EEG signals.

The main problems of epilepsy recognition by EEG are as follows: (1) Limited single feature leads to difficult extraction of signal feature. (2) Single layer neural network has limited ability to distinguish strong correlation signals. (3) Poor performance of single classifier has poor classification performance.

Thus, we carried out in-depth study on EEG signals of epilepsy. (1) Establish a multi-dimensional information fusion model. (2) The RBF model is improved to realize the accurate feature representation mechanism. (3) OAO strategy is introduced to carry out the research of classifiers to realize the recognition of epileptic EEG signals accurately.

## Algorithm

**Figure 1:**
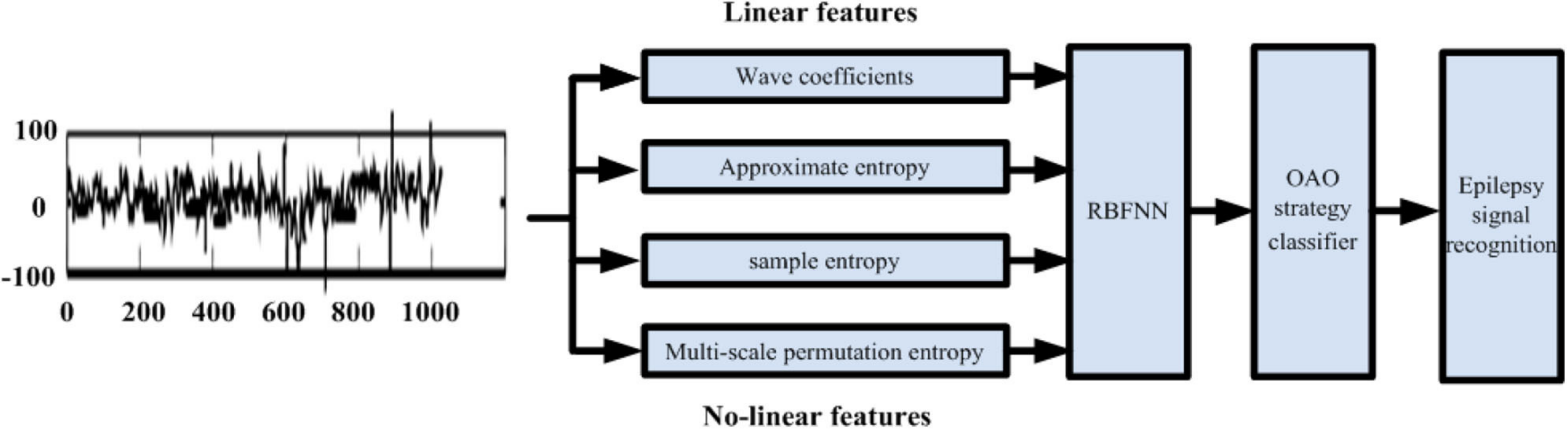
The algorithm flow chart.

According to above problems, we design the algorithm flow chart, as shown in Figure 1. First, EEG signal is input. Then, it is extracted from linear feature and non-linear feature. Wave coefficients are extracted from linear features. Approximate entropy, sample entropy and multi-scale permutation entropy are extracted from non-linear features to analyze from the energy point of view. On the basis of RBF, convolution neural network is constructed to extract signal features. OAO strategy classifier is established to recognize epileptic signals.

### Feature Extraction

Human brain signal contains linear information, mainly including time-domain, frequency-domain and time-domain analysis, which focus on EEG sequence waveform and amplitude statistics.

Wave coefficient *F*_*i*_(*n*) can measure the amplitude change of EEG sequence:

(1) $$\begin{array}{l} F_{i}\left(n\right)=\frac{1}{M-1}\overset{M-1}{\underset{j\;=\;1}{\sum }}|a_{n}\left(j+1\right)-a_{n}\left(j\right)| \end{array}$$

where *a*_*n*_ is the amplitude of n-th EEG data after wavelet transform; M is the signal length.

EEG signal has chaotic features. Only linear features of EEG signal cannot completely describe the signal. Therefore, we extract non-linear features of EEG sequence.

Entropy is used to express the uniformity of energy distribution in space. The more uniform the energy distribution, the greater the entropy. When the energy of a system is completely evenly distributed, the entropy of the system reaches the maximum (Longo, 2020). Thus, we measure the non-linear features of signal from the perspective of entropy.

Approximate entropy uses short data to distinguish different types of time series accurately. Considering different states of healthy period, epileptic intermittence period and epileptic period, the waveform, frequency, amplitude and other manifestations of EEG sequence are different, so this feature is used to identify EEG sequence.

Given there are *N* EEG data and similarity tolerance *N*. Approximate entropy *AE*(*m,r*) represents the probability that two sequences of adjacent m points are still adjacent after mapping to *m* +1 dimensional space on the basis of *r* in the original sequence of N sample points.

(2) $$\begin{array}{l} AE\left(m,r\right)=\phi^{m}\left(r\right)-\phi^{m+1}\left(r\right) \end{array}$$

(3) $$\{\begin{array}{l} \phi^{m}\left(r\right)=\frac{1}{N-m}\sum_{i\;=\;1}^{N-m}\ln C_{i}^{m}\left(r\right)\\ C_{i}^{m}\left(r\right)=\frac{Q}{N-m-1};\\ d_{ij}=\underset{k=0!\;m-1}{\max}\left[\left|x\left(i+k\right)-x\left(j+k\right)\right|\right] \end{array}$$

were ϕ^*m*^(*r*), $C_{i}^{m}\left(r\right)$, and *d*_*ij*_ are the intermediate variables.

Approximate entropy can express the similarity of signals, but it is difficult to explain the complexity of signals, which reduces the ability of analyzing problems. For this reason, sample entropy is introduced:

(4) $$\begin{array}{l} SE\left(m,r\right)=-\ln\;\frac{B^{m+1}\left(r\right)}{B^{m}\left(r\right)} \end{array}$$

(5) $$\{\begin{array}{l} B^{m}\left(r\right)=\frac{1}{N-m}\sum_{i\;=\;1}^{N-m}B_{i}^{m}\left(r\right)\\ B_{i}^{m}\left(r\right)=\frac{1}{N-m-1}\sum_{j\;=\;1,j\neq i}^{N-m}\ln\frac{Q}{N-m-1} \end{array}$$

where $B_{i}^{m}\left(r\right)$ is the energy contained in single signal.

Compared with approximate entropy, sample entropy eliminates the comparison of its own data, has strong independence and overcomes the dependence on other data; the consistency of sample entropy is good. When *m* and *r* are changed, the relationship of sample entropy size of different EEG sequences will not be changed; Compared with approximate entropy, sample entropy needs less data and even loses a part of data, which can still obtain reliable results.

Multiscale permutation entropy is improved on the basis of permutation entropy. The basic idea is to calculate multiscale coarse-grained time series, and then calculate permutation entropy. Suppose that the time series with length L is coarsely granulated as follows:

(6) $$y_{j}^{s}=\frac{1}{s}\sum_{i\;=\;\left(j-1\right)s+1}^{js}x_{i},\;j\in \left[1,L/s\;\right]$$

where *s* is the scale factor; $y_{j}^{s}$ is the multi-scale time series. When *s*=1, it is the original time series. The calculated entropy is the permutation entropy. After the multi-scale calculation of the visual sequence, the permutation entropy is estimated to calculate the multi-scale permutation entropy of the time sequence.

Time series $y_{j}^{s}$ is reconstructed as $Y_{t}^{s}=\left\{y_{t}^{s},y_{t+\tau }^{s},...y_{t+\left(m-1\right)\tau }^{s}\right\}$, where *m* is the embedding dimension, τ is the delay factor, and the pairs are arranged in ascending order. The probability of the permutation is calculated:

(7) $$\begin{array}{l} P_{l}^{s}=\frac{N_{l}}{n/s-m+1} \end{array}$$

Then the entropy of multiscale arrangement is:

(8) $$\begin{array}{l} H_{P}^{s}=\overset{m!}{\underset{l\;=\;1}{\sum }}P_{l}^{s}\ln\;P_{l}^{s} \end{array}$$

When $H_{P}^{s}$ reaches the maximum value, the permutation entropy is normalized:

(9) $$\begin{array}{l} h_{P}^{s}=H_{P}^{s}/\ln\;\left(m!\right) \end{array}$$

### Improved RBFNN

RBFNN (radial basis function neural network) has good generalization ability, and can approach the specified continuous function with any precision. When dealing with the classification problem, the linear non-separable problem in the original feature space is transformed into the linear separable problem in the high-dimensional feature space through the non-linear mapping of the hidden layer.

(10) $$\begin{array}{l} y=\varphi^{T}\left(\text{x}\right)\omega -b \end{array}$$

where *d* is the number of neurons; *b* is the threshold; **x** is the input vector; ω is the weight of the output layer.

(11) $$\{\begin{array}{l} \varphi \left(x\right)={\left[f_{1}\left(x\right),f_{2}\left(x\right),...f_{K}\left(x\right)\right]}^{T}\\ x={\left[x_{1},x_{2}...x_{d}\right]}^{T}\\ \omega ={\left[\omega_{1},\omega_{2}...\omega_{d}\right]}^{T} \end{array}$$

*f*_*k*_ represents the radial basis function of the k-th neuron in the hidden layer:

(12) $$\begin{array}{l} f_{k}\left(x\right)=\exp\left(-\frac{||x-c_{k}||}{2\sigma_{k}^{2}}\right) \end{array}$$

where *c*_*k*_ and σ_*k*_ are the center and width of radial basis function, respectively.

The minimax probability machine (MPM) is a binary classification model based on the minimization of upper bound of misclassification probability. It is defined as:

(13) $$\begin{array}{l} \underset{\alpha ,w\neq 0,b}{\max}\;\alpha ,\;s.t.\underset{x\sim \left(u_{+},\sum_{+}\right)}{\inf}\text{ pr}\left(w^{T}x\geq b\right)\geq \alpha ,\\ \underset{x:\left(u_{-},\sum_{-}\right)}{\inf}\text{ pr}\left(w^{T}x\leq b\right)\geq \alpha \end{array}$$

where $\underset{x\sim \left(u_{+},\sum_{+}\right)}{\inf}\text{pr}\left({\text{w}}^{T}\text{x}\geq b\right)$ represents the lower bound of probability when the condition is **w**^*T*^**x** ≥ *b*, *x*:(*u*_+_, ∑_+_). For the same reason, $\underset{x:\left(u_{-},\sum_{-}\right)}{\inf}\text{pr}\left({\text{w}}^{T}\text{x}\leq b\right)$.

For two kinds of data subject to different distributions, there is an optimal hyperplane (w^*^)^*T*^*x* = b^*^, which maximizes the lower bound α of the correct classification probability. When μ_+_ = μ_−_, by solving:

(14) $$\begin{array}{l} \gamma {\left(\alpha \right)}^{-1}=\underset{w}{\min}\left(\sqrt{w^{T}\sum_{+}w}+\sqrt{w^{T}\sum_{-}w}\right)\\ w^{T}\left(\mu_{+}-\mu_{-}\right)=1 \end{array}$$

The optimal solution is w^*^, then the optimal solution of b can be set as:

(15) $$\begin{array}{l} b^{*}={\left(w^{*}\right)}^{T}\mu_{+}-\gamma^{*}\left(\alpha \right)\sqrt{{\left(w^{*}\right)}^{T}\sum_{+}\left(w^{*}\right)} \end{array}$$

The lower bound of correct classification probability can be obtained by using the optimal solution:

(16) $$\begin{array}{l} \alpha^{*}=\frac{1}{1+{\left(\sqrt{{\left(w^{*}\right)}^{T}\sum_{+}\left(w^{*}\right)}+\sqrt{{\left(w^{*}\right)}^{T}\sum_{-}\left(w^{*}\right)}\right)}^{2}} \end{array}$$

The value of α fully reflects the separability between two types of data. When it is closer to 1, the classification model is more reliable. It indicates that the stronger the separability between two types of data.

Considering the equivalence of RBF neural network and TSK fuzzy system under certain conditions, the objective function is defined as:

(17) $$\begin{array}{r} \underset{\alpha ,w\neq 0,b}{\max}\;\alpha ,\;s.t.\underset{\psi \left(w\right)-\left(u_{\phi \left(+\right)},\sum_{\psi \left(+\right)}\right)}{\inf}\text{ pr}\left(w^{T}\psi \left(x\right)\geq b\right)\geq \alpha ,\\ \underset{\psi \left(w\right)-\left(u_{\phi \left(-\right)},\sum_{\psi \left(-\right)}\right)}{\inf}\text{ pr}\left(w^{T}\psi \left(x\right)\leq b\right)\geq \alpha \; \end{array}$$

It represents the corresponding vector of *x* in the new feature space obtained by RBF neural network mapping. The covariance of the mapped data samples can be estimated from the data sample set ψ (*x*).

(18) $$\begin{array}{l} \sum_{\psi }=\left(X_{\psi }-\mu_{\psi }\right){\left(X_{\psi }-\mu_{\psi }\right)}^{T}/N \end{array}$$

The optimization objective function is as follows:

(19) $$\begin{array}{l} \gamma {\left(\alpha \right)}^{-1}=\underset{w}{\min}\left(\sqrt{w^{T}\sum_{\psi \left(+\right)}w}+\sqrt{w^{T}\sum_{\psi \left(-\right)}w}\right)\\ w^{T}\left(\mu_{\psi \left(+\right)}-\mu_{\psi \left(-\right)}\right)=1 \end{array}$$

where α can describe the separability between two kinds of data and measure the reliability of classification model. According to the complexity of classification problem, by adjusting the number of neurons in hidden layer, the balance between the improvement of classification accuracy and the complexity of control model can be achieved.

One against one (OAO) strategy can resolve a complete multi-classification problem into multiple sub classification problems, and finally train the finite element classifier (Setiawan et al., 2020). Compared with one against rest (OAR) strategy, each subcategory is less difficult and easy to find a simple and effective interface to explain. The “voting method” is generally adopted, when OAO strategy test is applied. However, the problem of voting method is that the same number of votes of multiple classes will lead to the phenomenon of classification rejection, and each input data needs to be compared multiple times.

In order to avoid the classification rejection of voting method and improve the efficiency of classification model, we use exclusion method to build classification decision tree. Each internal node of the tree is a binary classifier, which means the method of exclusion along the direction of tree growth only needs *M*-1 comparison to get the classification results. In order to reduce the inherent “error accumulation” of tree structure, in this paper, we will make full use of the index provided by the minimum maximum probability technology. The binary classifier with large index has priority to do the classification with high assurance first. We normalize all signals and send them to RBFNN at a uniform scale. It is shown that the number of neurons is 1,024 and the size of nuclear function is 5 × 5 indicated through experiments and related references.

Training process:

1. Specify the number of neurons to get the center and width of each radial basis function.
2. Trained data is mapped to new feature space through RBF.
3. Train binary classifier with OAO strategy.
4. The classifier with the largest α is used as the root node of the classification tree.
5. If the classification result is that the sample does not belong to class *i*, then the available classifier of its child nodes is *C* = *C*\*I*, and the classifier used is the one with the largest index related to *j* in *C*;

Repeat all the process until traversing all child nodes.

## Experimental Results and Analysis

### Experiment Data and Experiment Platform

All data come from the epileptic EEG signal data experiment provided by the University of Bonn, Germany. This data set is divided into five groups. Each group of data contains 100 EEG signal segments of 23.6 s. Sampling frequency is 173.61 Hz with 4,097 sampling points, as shown in Table 1. All data come from the epileptic EEG signal data experiment provided by the University of Bonn, Germany. This data set is divided into five groups, where the ratio of training to testing is 1:1. We use different sampling evaluation rates to sample the sequence randomly and normalize it, which has increased the number of positive samples.

**Table 1:** Date set description.

**Sate**	**Serial number**	**Description**
Healthy	1	EEG signal when opening eyes
	2	EEG signal when closing eyes
Sick	3	EEG signal in hippocampus during intermission
	4	EEG signal in epileptic area during intermission
	5	EEG signal in the onset period

The experiment is based on WinXP, VC++ program and core dual core processor. Based on the above database, three groups of experiments are designed: DATA: EEG signals of the healthy and the sick are divided into two categories. Data 2: EEG signals of the healthy, the sick interval and the disease attack are divided into three categories. Data 3: each group of EEG signal is divided into one category.

### Feature Extraction Performance

Based on the traditional RBFNN, linear kernel, non-linear kernel and the proposed fusion kernel are compared. In the form of “mean ± standard deviation,” the test sensitivity (*SEN*), specificity (*SPE*) and accuracy (*ACC*) of each algorithm for data set classification are given:

(20) $$\begin{array}{l} SEN=\frac{TP}{TP+FN} \end{array}$$

(21) $$\begin{array}{l} SPE=\frac{TN}{TN+FP} \end{array}$$

(22) $$\begin{array}{l} ACC=\frac{TP+TN}{TP+FP+TN+FN} \end{array}$$

As well as the α index in this paper to measure the performance of feature extraction.

As shown in Tables 2–4, the linear and non-linear combined feature model proposed in this paper has achieved good results in all data sets. the α index is related to *SPE* and *SEN*, and is closely related to missed diagnosis rate and misdiagnosis rate in medical diagnosis. When using decision tree classification, the significance of choosing the classifier with large α index is to reduce the accumulation error. In Group 5, the signal is easy to recognize; α index is large; specificity is high and misdiagnosis rate is low. Therefore, we can distinguish the epileptic patients in the onset period, and then further diagnose whether the subjects are healthy or in the seizure interval. The proposed algorithm has strong non-linear classification ability, and has the advantages of simplicity, high efficiency and strong interpretation. In the training stage, we can find the differences of the distribution relations of all kinds of data, and build an effective classification tree model, without having to calculate the sensitivity and specificity in the test stage before doing comparative analysis.

**Table 2:** DATA 1 comparison of feature extraction performance.

**Index**	**Linear kernel**	**Non-linear kernel**	**Fusion kernel**
*SPE*	0.802 ± 0.042	0.932 ± 0.023	0.951 ± 0.021
*SEN*	0.841 ± 0.019	0.946 ± 0.019	0.965 ± 0.047
*ACC*	0.843 ± 0.021	0.932 ± 0.024	0.963 ± 0.024
*α(*(1, 2):(3, 4, 5))	0.456 ± 0.012	0.951 ± 0.031	0.963 ± 0.013

**Table 3:** DATA 2 comparison of feature extraction performance.

**Index**	**Linear kernel**	**Non-linear kernel**	**Fusion kernel**
*SPE*	0.946 ± 0.022	0.945 ± 0.013	0.963 ± 0.009
	0.973 ± 0.026	0.956 ± 0.022	0.923 ± 0.015
	0.932 ± 0.012	0.962 ± 0.013	0.912 ± 0.009
*SEN*	0.943 ± 0.045	0.965 ± 0.032	0.951 ± 0.018
	0.890 ± 0.055	0.935 ± 0.021	0.953 ± 0.037
	0.973 ± 0.048	0.942 ± 0.034	0.910 ± 0.056
*ACC*	0.918 ± 0.019	0.943 ± 0.013	0.960 ± 0.024
α((1,2):(3,4))	0.813 ± 0.015	0.702 ± 0.007	0.775 ± 0.001
α((1,2):(5))	0.951 ± 0.014	0.762 ± 0.011	0.973 ± 0.001
α((3,4):(5))	0.926 ± 0.009	0.783 ± 0.023	0.949 ± 0.003

**Table 4:** DATA 3 comparison of feature extraction performance.

**Index**	**Linear kernel**	**Non-linear kernel**	**Fusion kernel**
*SPE*	0.963 ± 0.012	0.971 ± 0.023	0.944 ± 0.031
	0.981 ± 0.021	0.961 ± 0.024	0.983 ± 0.015
	0.933 ± 0.026	0.923 ± 0.017	0.893 ± 0.051
	0.931 ± 0.024	0.901 ± 0.012	0.919 ± 0.048
	0.987 ± 0.013	0.961 ± 0.032	0.998 ± 0.061
*SEN*	0.791 ± 0.031	0.810 ± 0.062	0.910 ± 0.063
	0.921 ± 0.042	0.891 ± 0.120	0.823 ± 0.056
	0.581 ± 0.123	0.651 ± 0.130	0.713 ± 0.166
	0.589 ± 0.120	0.661 ± 0.067	0.613 ± 0.114
	0.981 ± 0.031	0.953 ± 0.035	0.865 ± 0.067
*ACC*	0.813 ± 0.036	0.769 ± 0.035	0.784 ± 0.056
α(1:2)	0.753 ± 0.015	0.621 ± 0.022	0.701 ± 0.031
α(1:3)	0.856 ± 0.007	0.765 ± 0.012	0.758 ± 0.007
α(2:3)	0.920 ± 0.006	0.841 ± 0.010	0.895 ± 0.008
α(1:4)	0.821 ± 0.013	0.761 ± 0.010	0.776 ± 0.031
α(2:4)	0.901 ± 0.006	0.812 ± 0.005	0.893 ± 0.008
α(3:4)	0.381 ± 0.031	0.273 ± 0.032	0.351 ± 0.033
α(1:5)	0.978 ± 0.003	0.813 ± 0.013	0.983 ± 0.003
α(2:5)	0.953 ± 0.004	0.790 ± 0.012	0.972 ± 0.012
α(3:5)	0.957 ± 0.009	0.789 ± 0.013	0.968 ± 0.005
α(4:5)	0.913 ± 0.011	0.743 ± 0.018	0.912 ± 0.007

### Performance Comparison of Classification Algorithms

**Figure 2:**
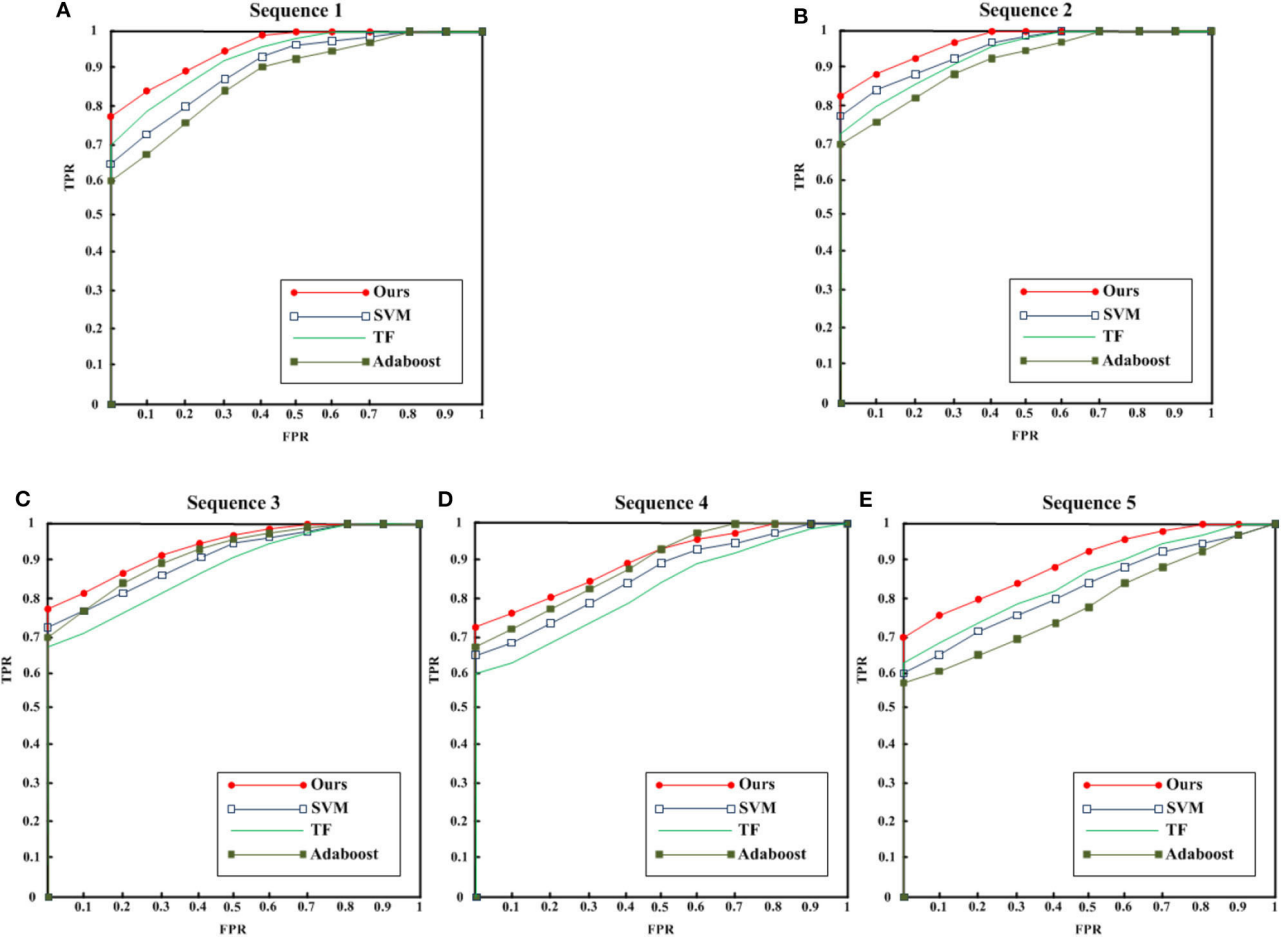
ROC curves. **(A)** Sequence 1, **(B)** sequence 2, **(C)** sequence 3, **(D)** sequence 4, and **(E)** sequence 5.

We use different algorithms to statistic ROC curves of all data, as shown in Figure 2. Generally, the proposed algorithm achieve better effect. We use the corresponding algorithm in the references. Based on the particularity of the EEG signal and the parameters setting mentioned in the references, we perform fine tuning experiment to ensure the performance of the proposed algorithm. As shown in Figures 2A,B, all algorithms for healthy EEG signals have achieved good results. Due to the multi-scale nature of AdaBoost algorithm cannot fully show the features of EEG signal, the detection effect is slightly low. For the EEG signal Figures 2C–E, during the onset, the detection effect is lower than that of healthy EEG signal because of the short onset time and limited display of EEG signal. SVM (Murugavel and Ramakrishnan, 2016) is classified by time-domain features. TF (time frequency) (Sharma and Pachori, 2017) algorithm establishes the relationship between time and frequency for analysis. Andrzejak et al. (2001) establishes a multi-scale classification framework. Because of the classification tree structure used in the decision-making stage, the proposed method is simple and efficient. Compared with traditional algorithms, the proposed method combines RBF neural network, so it has better non-linear approximation ability and generalization performance to achieve the best detection effect.

## Conclusions

Aiming at the difficulty of classification of epileptic EEG signals, this paper analyzes the problem from the feature level, and proposes the feature structure combining linearity and non-linearity. In order to better represent the epileptic signal, the RBF algorithm is improved, and the one-again-one (OAO) strategy is introduced to realize the classification of epileptic signal by computer means, which is better than the current mainstream algorithm.

## Data Availability Statement

The raw data supporting the conclusions of this article will be made available by the authors, without undue reservation.

## Author Contributions

All authors listed have made a substantial, direct and intellectual contribution to the work, and approved it for publication.

## Conflict of Interest

The authors declare that the research was conducted in the absence of any commercial or financial relationships that could be construed as a potential conflict of interest.
